# Delphinidin modulates neuroinflammation and behavioral deficits in a Parkinson’s disease mouse model

**DOI:** 10.1038/s41531-025-01244-0

**Published:** 2026-01-12

**Authors:** A. Grotemeyer, S. Alexander, L. Frieß, J. Roewer, E. E. Bankoglu, M. Badr, J. Wu, H. Stopper, J. Volkmann, N. Roewer, C. W. Ip

**Affiliations:** 1 Department of Neurology, University Hospital of Wuerzburg, Würzburg, Germany; 2 Dermatologikum Hamburg, Hamburg, Germany; 3 Institute of Pharmacology and Toxicology, University of Wuerzburg, Würzburg, Germany; 4 Zentrum für Operative Medizin (Chirurgie I), University Hospital of Wuerzburg, Würzburg, Germany

**Keywords:** Immunology, Neurology, Neuroscience

## Abstract

Neuroinflammation is deeply intertwined with dopaminergic (DA) neurodegeneration in Parkinson’s disease (PD). We tested whether delphinidin, an anthocyanidin with reported inflammasome/NF-κB modulatory activity, alters neuroinflammation and nigrostriatal integrity in a progressive AAV1/2-A53T α-synuclein (hαSYN) mouse model. Once-daily intraperitoneal delphinidin for nine weeks modestly ameliorated asymmetric forepaw use, attenuated the hαSYN-induced loss of striatal TH⁺ terminal density, and was associated with modest alterations in dopamine turnover, yet did not prevent the loss of DA neurons in the substantia nigra (SN). On the immunological level, delphinidin attenuated the innate immune response by reducing the number and activity of CD11b^+^ microglia in both the SN and striatum. In contrast, CD4^+^-mediated adaptive inflammation remained unchanged, while the number of CD8^+^ T cells increased in the SN. Notably, approximately 48% of CD8^+^ T cells in the SN of these mice were identified as CD8^+^CD122^+^ regulatory T cells, known for their anti-inflammatory properties. In conclusion, delphinidin was associated with a partial attenuation of neuroinflammatory changes and a context-dependent shift towards a more anti-inflammatory CD8⁺CD122^+^ T cell phenotype in the SN. However, these changes did not translate into protection of SN DA somata, revealing a dissociation between striatal terminal preservation and nigral cell body survival, and underscoring the limitations of targeting innate immunity alone under the current dosing paradigm.

## Introduction

Parkinson’s disease (PD) is a frequent neurodegenerative disorder with a close relationship to neuroinflammation^1^. Dopaminergic (DA) cell death is interwoven with accumulation of pathological α-synuclein aggregates^2^ and increased pro-inflammatory activity of both—the innate and adaptive immune system^1,3–5^. The NLRP3 (NOD-, LRR-, and pyrin domain-containing protein 3) inflammasome is a critical component of the innate immune system, functioning as a sensor for pathogenic microbes and cellular stress signals. Upon activation, it orchestrates the release of key pro-inflammatory cytokines, including interleukin-1β (IL-1β), thereby initiating and amplifying immune responses to infection and tissue damage^6^. Direct inhibition of the NLRP3 inflammasome using selective agents, such as the small-molecule inhibitor MCC950, significantly reduces (neuro)inflammation and protects from cell death in various (inflammatory) diseases^7–10^. This intervention has demonstrated efficacy across multiple neurodegenerative disease models^9,11^, emphasizing its potential therapeutic impact specifically for PD by mitigating neuroinflammatory cascades that drive neuronal loss^12–14^. However, given the potential adverse effects associated with selective NLRP3 inhibition, including immunosuppressive side effects^15^, there is a compelling rationale for exploring alternative compounds. Such alternatives should aim to provide safer and more tolerable therapeutic options, emphasizing their anti-inflammatory and neuroprotective potential.

Anthocyanins, including polyphenols such as delphinidin derived from plant extracts and thus naturally occurring, are under investigation for their potential to modulate the NLRP3 inflammasome^16,17^. While there is no specific literature confirming their safety in this context, existing studies on polyphenols suggest they are generally well-tolerated, allowing for a reasonable assumption of good safety^16–19^. In addition, delphinidin exerts its anti-inflammatory effect by inhibiting the activation and nuclear translocation of nuclear factor kappa-light-chain-enhancer of activated B cells (NFκB), a key transcription factor involved in the regulation of immune and inflammatory responses^20–22^. Selective inhibition of NFκB was shown to protect 1-methyl-4-phenyl-1,2,3,6-tetrahydropyridine (MPTP) PD mice from DA cell death^23^.

The objective of this study was to determine whether attenuation of the innate immune response by delphinidin could function as a therapeutic agent for the treatment of PD. This investigation is based on the hypothesis that polyphenols may possess the potential to slow or halt the progression of the disease^18,24^. To evaluate this, we utilized the AAV-1/2-A53T-α-synuclein mouse model (hαSYN), in which AAV-1/2-A53T-α-synuclein was unilaterally injected directly in the substantia nigra (SN) to establish a progressive PD model that closely mimics the pathophysiology of human PD^3,12,25,26^. Notably, this model faithfully recapitulates key aspects of human PD neuropathology, particularly the involvement of CD8^+^ and CD4^+^ cells in the progression of neurodegenerative processes over time^3^.

Our findings demonstrate that intraperitoneal (i.p.) administration of delphinidin reduces innate immune activity in hαSYN mice, evidenced by decreased CD11b^+^ microglial activation, while increasing the number of anti-inflammatory CD8^+^ regulatory T cells. Furthermore, delphinidin treatment modestly ameliorated motor deficits, attenuated dopaminergic fiber loss, and was associated with altered dopamine turnover in the striatum, but it did not prevent dopaminergic cell loss in the SN. This study underscores the critical role of immune cells in neuroinflammation-driven DA cell death in PD^3,12,27^ and emphasizes the urgent need for further investigation into effective and well-tolerated anti-inflammatory agents as potential therapeutic options for human PD.

## Results

### Biological half-life of i.p. administered delphinidin

**Fig. 1:**
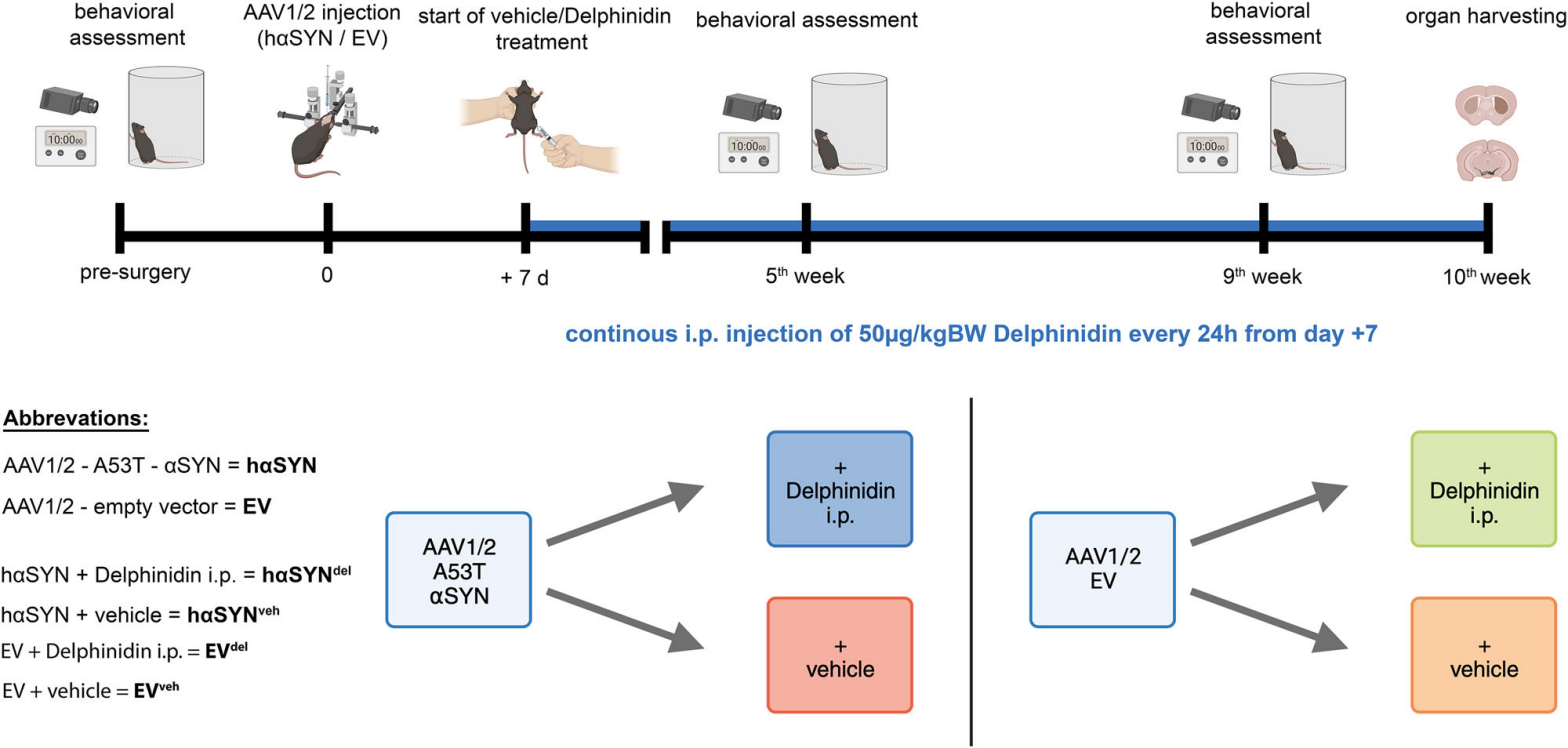
Illustration of the experimental design. Timeline showing timepoints of behavioral testing, AAV1/2 injection, and start of delphinidin i.p. treatment (+7 days after viral vector injection). Organ harvesting was performed after 10 weeks of the experiment. created with Biorender.com.

**Fig. 2:**
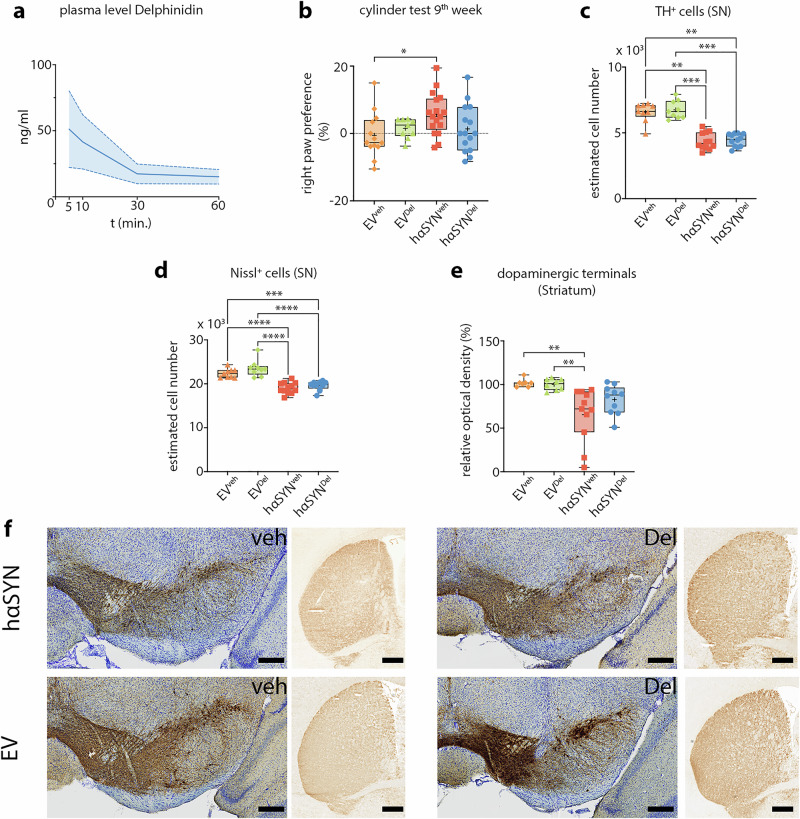
Delphinidin attenuates hαSYN-induced motor deficits, reduces dopaminergic fiber loss but does not prevent loss of dopaminergic SN neurons. **a** Assessment of plasma delphinidin levels after i.p. administration. For every indicated time point independent cohorts of animals were used (n = 4 / time point). **b** Evaluation of motor performance in the cylinder test. Statistical analysis by one-way ANOVA followed by Tukey’s multiple comparisons test: F(3,50) = 2.753, P = 0.0522, n-numbers: EV^veh^ = 13, EV^del^ = 9, hαSYN^veh^ = 17, hαSYN^del^ = 15 **c** Estimated cell count of TH^+^ cells in the SN. Statistical analysis by Kruskal–Wallis test followed by Dunn’s multiple comparisons test: Kruskal–Wallis statistic = 28.30, P <0.0001, n-numbers: EV^veh^ = 9, EV^del^ = 9, hαSYN^veh^ = 12, hαSYN^del^ = 12; significance: hαSYN^veh^ compared to EV^veh^: ~33.4%, P = 0.0015 and EV^del^: ~35.2%, P = 0.0005; hαSYN^del^ to EV^veh^: ~32.0%, P = 0.0022 and EV^del^: ~33.9%, P = 0.0007. **d** Estimated cell count of Nissl^+^ cells in the SN. Statistical analysis by one-way ANOVA followed by Tukey’s multiple comparisons test: F(3, 38) = 28.33, P <0.0001, n-numbers: EV^veh^ = 9, EV^del^ = 9, hαSYN^veh^ = 12, hαSYN^del^ = 12; significance: hαSYN^veh^ compared to EV^veh^: ~14.9%, P <0.0001 and EV^del^: ~18.9%, P <0.0001; hαSYN^del^ compared to EV^del^: ~12.5%, P = 0.0001 and EV^del^: ~16.6%, P <0.0001 **e** Relative optical density of TH^+^ terminals in the striatum. Statistical analysis by Kruskal–Wallis test followed by Dunn’s multiple comparisons test: Kruskal–Wallis statistic = 19.63, P <0.0001, n-numbers: EV^veh^ = 7, EV^del^ = 8, hαSYN^veh^ = 11, hαSYN^del^ = 10; significance: hαSYN^veh^ compared to EV^veh^: P = 0.0019 and EV^del^: P = 0.0029 **f** Representative RGB color images of right SN and striatum for each group. Scale bars: 250 µm (SN) and 500 µm (striatum). *P <0.05, **P <0.01, ***P <0.001, ****P <0.0001. Data are shown as min-to-max and mean (+).

We first assessed the biological half-life of delphinidin following i.p. injection (Fig. 1) into 10-week-old B6/J wildtype mice at multiple time points (Fig. 2a). Each time point represents 4 individual mice that were sacrificed at 5, 10, 30, and 60 min after i.p. Delphinidin administration. We found that systemic administration of delphinidin resulted in a peak plasma concentration within 5 min (51.175 ng/ml ± 28.974 SEM, n = 4) and showed a decrease within the next 5 min (41.550 ng/ml ± 20.504 SEM, n = 4) and a further sharp decrease within 30 min (17.375 ng/ml ± 7.459 SEM, n = 4). Finally, the plasma level remained stable until the end of the observation interval (60 min) (15.15 ng/ml ± 5.464 SEM, n = 4) (Fig. 2a).

### Delphinidin attenuates hαSYN-induced motor deficits and reduces dopaminergic fiber loss in the striatum of hαSYN mice

After characterizing the pharmacokinetics of delphinidin, we employed the cylinder test to investigate the impact of daily i.p. administration of delphinidin on PD-like behavior in hαSYN mice over a period of 9 weeks. A significant increase in right paw preference was observed in vehicle-treated hαSYN (hαSYN^veh^) compared to vehicle-treated AAV1/2-empty vector (EV^veh^) mice (1.12-fold, P = 0.0430; Fig. 2b), indicative for an impaired motor performance in the PD model. In contrast, delphindin-treated hαSYN (hαSYN^del^) mice demonstrated comparable motor performance as EV^veh^ mice, suggesting an amelioration of asymmetric forepaw use. Although the direct comparison between hαSYN^del^ and hαSYN^veh^ mice did not reach statistical significance, the data indicate a trend towards amelioration of the hαSYN-induced motor deficit (Fig. 2b).

Due to the diminished PD-like behavior observed in hαSYN^del^ mice, we proceeded to investigate the effects of delphinidin on TH^+^ DA neurons in the SN (Fig. 2c). Interestingly, the total number of TH^+^ cells was significantly decreased in both hαSYN^veh^ (compared to EV^veh^: ~33.4% and EV^del^: ~35.2%) and hαSYN^del^ (compared to EV^veh^: ~32.0% and EV^del^: ~33.9%) mice compared to controls. Similarly, Nissl^+^ total neuronal cell counts were reduced in hαSYN^veh^ (compared to EV^veh^: ~14.9%, and EV^del^: ~18.9%) and hαSYN^del^ (compared to EV^veh^: ~12.5% and EV^del^: ~16.6%) mice (Fig. 2d, f). No significant differences in TH^+^ and Nissl^+^ cell numbers were observed comparing hαSYN^del^ and hαSYN^veh^ mice. However, the relative optical density (OD), reflecting the integrity of dopaminergic terminals in the striatum, showed a significant decrease only in hαSYN^veh^ mice compared to controls. In contrast, delphinidin-treated hαSYN^del^ mice exhibited less loss of dopaminergic fibers, with no significant difference from control groups (Fig. 2e). hαSYN^del^ mice do not show significant dopaminergic terminal loss compared to that seen in haSYN^veh^. These data suggest that delphinidin therapy attenuates motor deficits as well as the hαSYN-induced loss of striatal dopaminergic fibers.

### Delphinidin induces higher dopamine turnover in the striatum of hαSYN PD mice

**Fig. 3:**
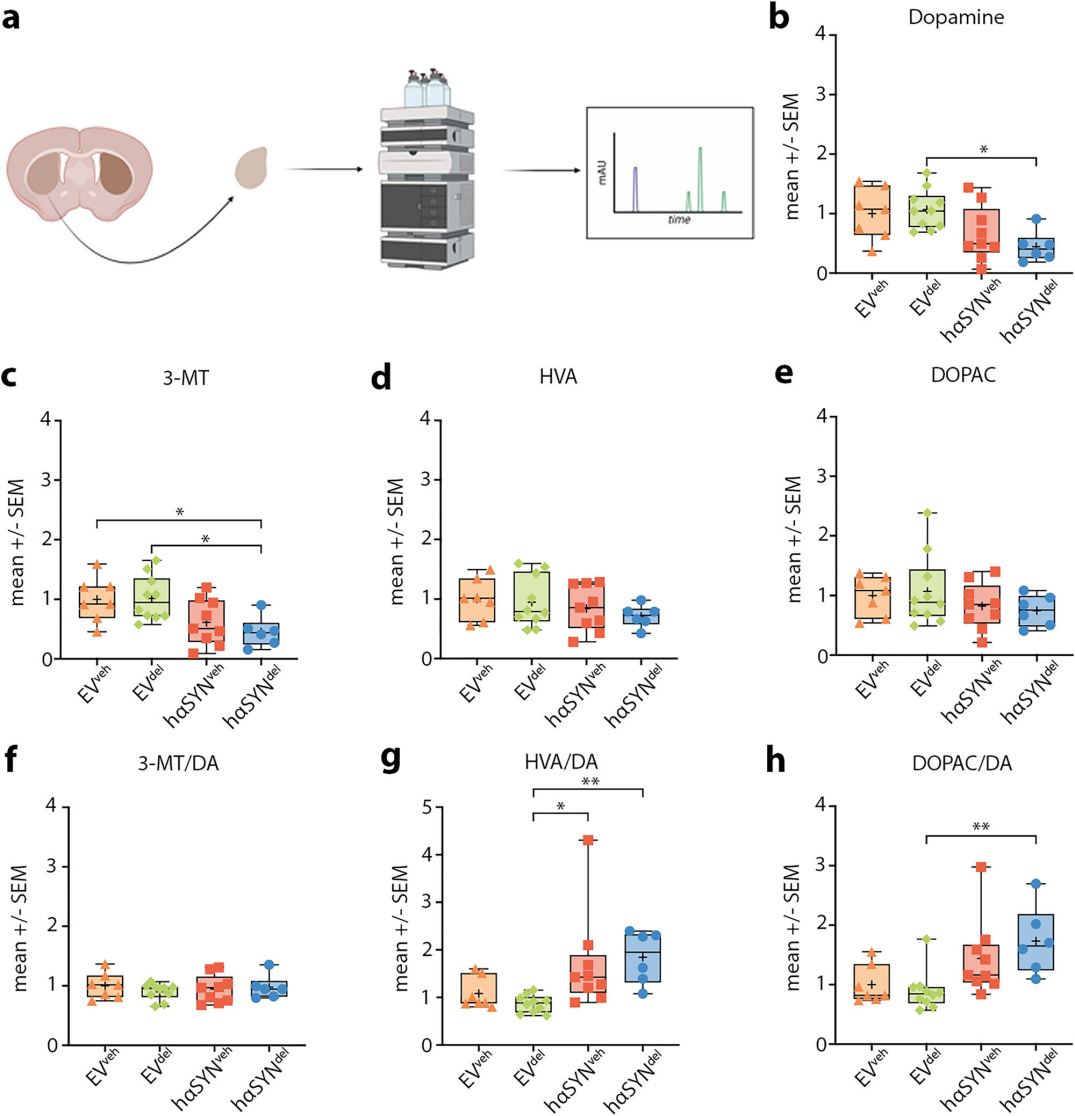
Delphinidin modestly alters dopamine turnover in the striatum of hαSYN PD mice. **a** Sketch of HPLC processing, usage of ipsilateral striatum (created with biorender.com) **b** Comparison of normalized dopamine levels in the frontal striatum. Statistical analysis by one-way ANOVA followed by Tukey’s multiple comparisons test: F(3, 28) = 4.345, P = 0.0124 **c** Comparison of normalized 3-MT levels in the frontal striatum. Statistical analysis by one-way ANOVA followed by Tukey’s multiple comparisons test: F(3, 28) = 4.650, P = 0.0093 **d** Comparison of normalized HVA levels in the frontal striatum. Statistical analysis by one-way ANOVA followed by Tukey’s multiple comparisons test: F(3, 28) = 0.8402, P = 0.4833 **e** Comparison of normalized DOPAC levels in the frontal striatum. Statistical analysis by one-way ANOVA followed by Tukey’s multiple comparisons test: F(3, 28) = 0.9164, P = 0.4457 **f** Comparison of normalized 3-MT/DA turnover in the frontal striatum. Statistical analysis by Kruskal–Wallis test followed by Dunn’s multiple comparisons test: Kruskal–Wallis statistic = 0.8624, P = 0.8344 **g** Comparison of normalized HVA/DA turnover in the frontal striatum. Statistical analysis by Kruskal–Wallis test followed by Dunn’s multiple comparisons test: Kruskal–Wallis statistic = 15.99, P = 0.0011 **h** Comparison of normalized DOPAC/DA turnover in the frontal striatum. Statistical analysis by Kruskal–Wallis test followed by Dunn’s multiple comparisons test: Kruskal–Wallis statistic = 13.96, P = 0.0030. n-numbers: EV^veh^ = 7, EV^del^ = 10, hαSYN^veh^ = 9, hαSYN^del^ = 6. *P <0.05, **P <0.01, ***P <0.001, ****P <0.0001. Data are shown as min-to-max and mean (+).

To investigate whether the rescue of dopaminergic terminals by delphinidin treatment in hαSYN PD mice is associated with changes in dopamine metabolism, we next examined the levels of dopamine and its metabolites in the striatum (Fig. 3a). We observed a decrease of dopamine and 3-methoxytyramine (3-MT) levels when comparing hαSYN with EV mice, although no significant differences were found in DOPAC and HVA levels (Fig. 3b–e). Given the relevance of dopamine turnover to motor abilities, we further evaluated this aspect by calculating the 3-MT/DA, HVA/DA, and DOPAC/DA ratio (Fig. 3f–h). We found the 3-MT/DA turnover to be unchanged on the group level (Fig. 3f). However, while the HVA/DA ratio was elevated in both hαSYN^veh^ and hαSYN^del^ mice, the DOPAC/DA ratio revealed significantly increased DA turnover in hαSYN^del^ mice compared to EV^del^, but not in hαSYN^veh^ PD mice (Fig. 3g, h). No difference was observed comparing the dopamine turnover of hαSYN^del^ and hαSYN^veh^ mice. This finding suggests that dopamine turnover is selectively, but only modestly, altered in delphinidin-treated hαSYN mice.

### Delphinidin attenuates CD11b^+^ microglia activation in the SN and striatum of hαSYN mice

**Fig. 4:**
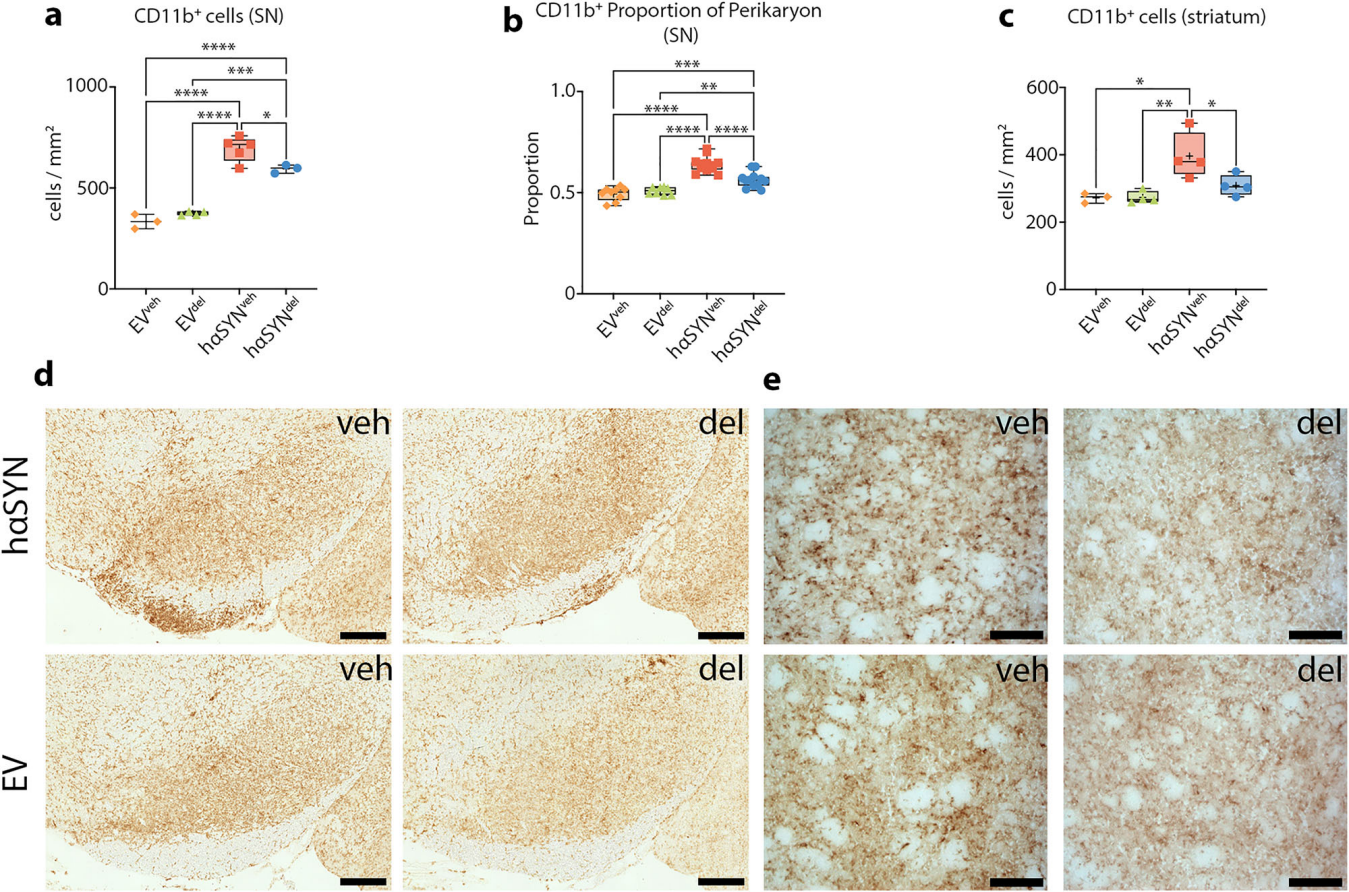
Delphinidin attenuates CD11b^+^ activity. **a** Quantification of CD11b^+^ microglia in the SN. Statistical analysis by one-way ANOVA followed by Tukey’s multiple comparisons test: F(3, 11) = 69.27, P <0.0001, n-numbers: EV^veh^ = 3, EV^del^ = 4, hαSYN^veh^ = 5, hαSYN^del^ = 3 **b** Cell size indicated by the perikaryon to nucleus proportion of CD11b^+^ microglia. Statistical analysis by one-way ANOVA followed by Tukey’s multiple comparisons test: F(3, 37) = 38.68, P <0.0001, n-numbers: EV^veh^ = 9, EV^del^ = 8, hαSYN^veh^ = 12, hαSYN^del^ = 12. **c** Quantification of CD11b^+^ microglia in the striatum. Statistical analysis by one-way ANOVA followed by Tukey’s multiple comparisons test: F(3, 11) = 7.696, P = 0.0048, n-numbers: EV^veh^ = 3, EV^del^ = 4, hαSYN^veh^ = 4, hαSYN^del^ = 4 **d** Representative RGB color images of right SN for each group (IHC staining). Scale bars: 250 µm **e** Representative RGB color images of right striatum (inset) for each group. Scale bars: 100 µm. *P <0.05, **P <0.01, ***P <0.001, ****P <0.0001. Data are shown as min-to-max and mean (+).

Given the known immune-modulatory effect of delphinidin, in particular on the innate immune system, we analyzed microglia activation in hαSYN^del^ mice and controls 10 weeks after AAV injection. Specifically, we assessed the absolute number of CD11b^+^ microglia cells in the SN and striatum, as well as microglial cell size in the SN. The absolute number of microglia in hαSYN^veh^ mice was significantly increased compared to both EV control groups (Fig. 4a), indicative for the pro-inflammatory environment in PD mice. Delphindin-treated hαSYN^del^ mice showed significantly reduced microglia number compared to hαSYN^veh^ mice in the SN (~14.1%, P = 0.0331; Fig. 4a, d). This reduction of microglial activity was further demonstrated by the analysis of cell size, assessed by the cell soma to nucleus ratio. The hαSYN^del^ mice exhibited a significantly smaller microglial cell size compared to hαSYN^veh^ mice (~11.8%, P <0.0001; Fig. 4b).

In line with this, reduced microglia number was also found in the striatum (~22.1%, P = 0.0498; Fig. 4c, e).

These findings suggest that a 9-week administration of delphinidin affects the innate immune system in hαSYN mice by reducing microglial activity in the nigrostriatal tract.

### Delphinidin promotes an anti-inflammatory effect by shifting the T cell response towards an anti-inflammatory phenotype

**Fig. 5:**
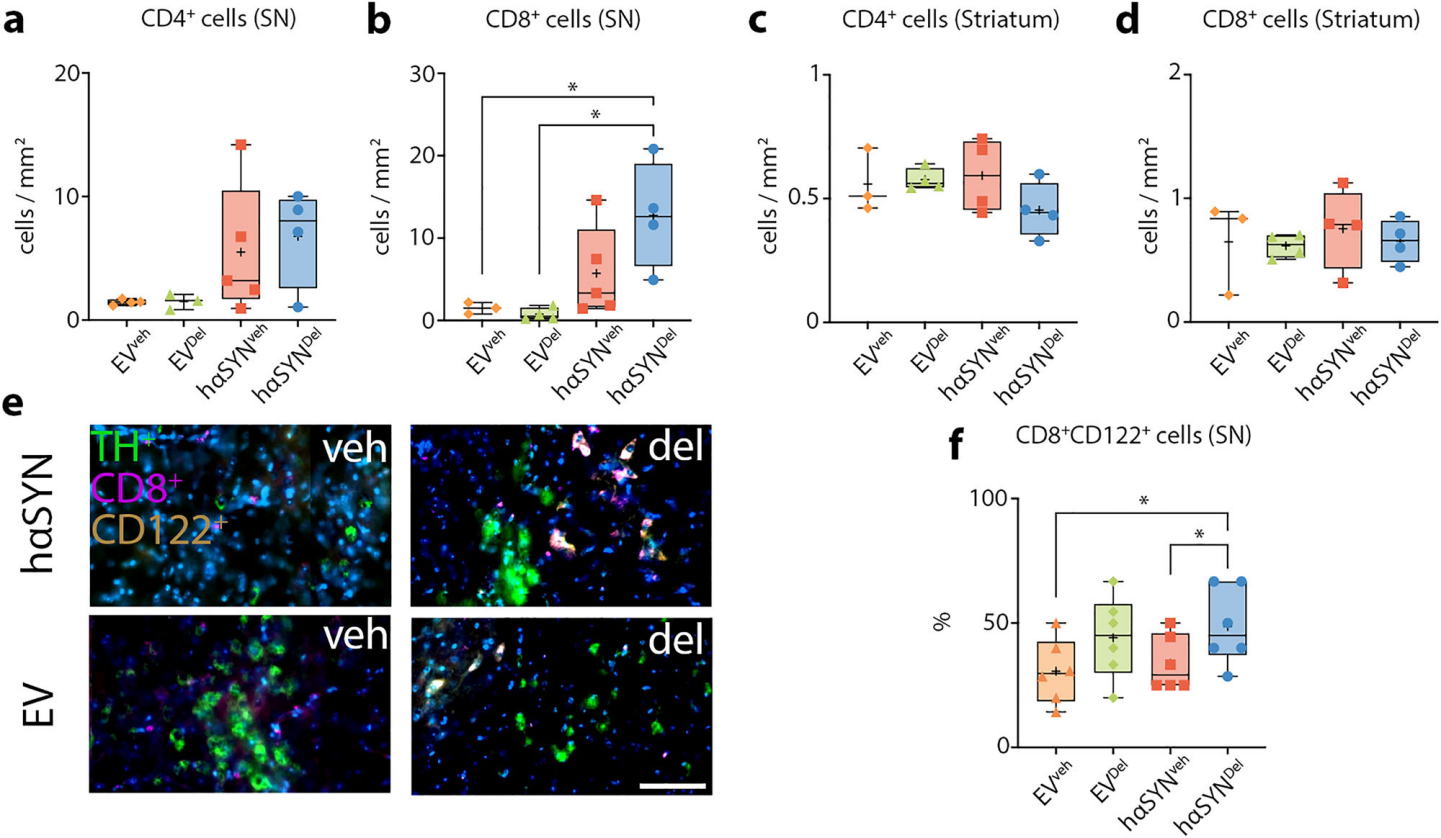
Delphinidin promotes an anti-inflammatory T cell response. **a** Quantification of CD4^+^ cells in the SN. Statistical analysis by one-way ANOVA followed by Tukey’s multiple comparisons test: F(3, 12) = 2.156, P = 0.1464, n-numbers: EV^veh^ = 3, EV^del^ = 4, hαSYN^veh^ = 5, hαSYN^del^ = 4 **b** Quantification of CD8^+^ cells in the substantia nigra (SN). Statistical analysis by one-way ANOVA followed by Tukey’s multiple comparisons test: F(3, 12) = 5.524, P = 0.0129, n-numbers: EV^veh^ = 3, EV^del^ = 4, hαSYN^veh^ = 5, hαSYN^del^ = 4 **c** Quantification of CD4^+^ cells in the striatum. Statistical analysis by one-way ANOVA followed by Tukey’s multiple comparisons test: F(3, 11) = 1.215, P = 0.3501, n-numbers: EV^veh^ = 3, EV^del^ = 4, hαSYN^veh^ = 4, hαSYN^del^ = 4 **d** Quantification of CD8^+^ cells in the striatum. Statistical analysis by one-way ANOVA followed by Tukey’s multiple comparisons test: F(3, 11) = 0.2133, P = 0.8851, n-numbers: EV^veh^ = 3, EV^del^ = 4, hαSYN^veh^ = 4, hαSYN^del^ = 4 **e** Representative IF images of CD8^+^CD122^+^ cells in the SN. Scale bar: 50 µm **f** Quantification of CD8^+^CD122^+^ cells in the SN – as indicated by TH^+^ cells. Statistical analysis by one-tailed multiple t-tests, only showing significant results, P = 0.0425 (hαSYN^veh^ vs. hαSYN^del^), P = 0.0270 (EV^veh^ vs. hαSYN^del^), n-numbers: EV^veh^ = 6, EV^del^ = 6, hαSYN^veh^ = 6, hαSYN^del^ = 6; *P <0.05, **P <0.01, ***P <0.001, ****P <0.0001. Data are shown as min-to-max and mean (+).

Comparison of CD4^+^ T cell infiltration revealed no significant differences among the groups; however, hαSYN^del^ mice demonstrated a trend towards increased CD4^+^ T cell numbers compared to hαSYN^veh^ mice (1.23-fold, P > 0.05; Fig. 5a). Notably, the SN of hαSYN^del^ mice showed a significant increase in CD8^+^ T cells compared to controls (EV^del^: 16.58-fold, P = 0.0140; EV^veh^: 8.56-fold, P = 0.0325; Fig. 5b). Although CD8^+^ T cells were elevated in hαSYN^veh^ mice compared to controls (EV^del^: 7.45-fold, P > 0.05; EV^veh^: 3.85-fold, P > 0.05), these differences were not significant. Similarly, the higher infiltration of CD8^+^ T cells into the SN of hαSYN^del^ mice compared to hαSYN^veh^ mice did not reach statistical significance (2.22-fold, P > 0.05). There was no evidence of elevated or attenuated T cell infiltration into the striatum between controls and hαSYN mice (CD4^+^: P > 0.05 for all groups; CD8^+^: P > 0.05 for all groups; Fig. 5c, d). Thus, delphinidin does not markedly change the overall magnitude of T cell infiltration. However, the higher CD8^+^ T cell infiltration in the SN of hαSYN^del^ animals could be attributed to a significantly increased proportion of CD8^+^CD122^+^ cells (~43.9%, P = 0.0425 (hαSYN^veh^ vs. hαSYN^del^); ~58.9%, P = 0.0270 (EV^veh^ vs. hαSYN^del^). This finding suggests a delphinidin-driven alteration in T cell response towards a more anti-inflammatory CD8^+^CD122^+^ phenotype (Fig. 5e, f).

## Discussion

Neuroinflammation is widely recognized as a main driver of DA neurodegeneration in PD, linking microglial activation and adaptive immunity to the progressive loss of SN neurons^4,5,27–29^. Classical observations of activated glia and infiltrating lymphocytes in postmortem PD brains^4,5,28^ and more recent mechanistic analysis’^3,12,14,27,29^ have verified the concept that neuroinflammation is disease-shaping. Despite this, available treatments remain still purely symptomatic, and no approved anti-inflammatory therapy has been shown to slow progression^30^.

Recent studies have highlighted the NLRP3-inflammasome as a critical innate immune node. The selective NLRP3 inhibitor MCC950, for instance, reduces cytokine release, preserves dopaminergic neurons, and improves motor behavior in PD models^12,14,31^. These data support the principle that inflammasome modulation can modify the disease trajectory. Consequently, we investigated the anti-inflammatory and neuroprotective properties of delphinidin, a naturally occurring anthocyanidin, as a possible therapeutic candidate for PD. Polyphenols of this class interact with inflammasome and NF-κB signaling^15–17,20,21^ and can be regarded as therapeutic candidates for clinical use^18^. Within in this study we observed distinct effects on the innate and adaptive immune system. On the innate axis, delphinidin reduced microglial number and soma size, in both, SN and striatum, consistent with suppressed pro-inflammatory signaling. On the adaptive axis, delphinidin shifted the CD8⁺ compartment towards a regulatory phenotype, with nearly half of infiltrating T cells expressing CD122.

Mechanistically, NF-κB acts upstream of the NLRP3 inflammasome by inducing the transcription of inflammasome components and (pro)-IL-1β^32,33^. Activation of this axis amplifies innate responses and promotes microglial cytokine release, which can facilitate recruitment and activation of T cells^34–36^. In contrast, the expansion of CD8⁺CD122⁺ T_reg_ cells under delphinidin is unlikely to result directly from NF-κB/NLRP3 modulation. These cells depend on IL-2/IL-15 signaling via the CD122 receptor^37,38^ and their proliferation may be indirectly favored by a less inflammatory milieu. As delphinidin has already been shown to suppress NF-κB and enhance anti-inflammatory cytokines such as IL-10 and TGF-β^39^, this could reduce effector competition for IL-2 and lead to regulatory CD8⁺ subset expansion.

Importantly, the principle that T_reg_ cells counteract neuroinflammation is well established. CD4⁺ T_reg_ attenuate neuronal loss^40^, and pharmacological expansion with CD28 superagonists or low-dose IL-2 exhibits neuroprotective properties^41–43^. Our observation that delphinidin expands CD8⁺CD122⁺ T_reg_ complements these findings and suggests that both CD4⁺ and CD8⁺ subsets act synergistically to restrain inflammation, mirroring the cooperation of their pro-inflammatory counterparts^1,3,44^. To our knowledge, no previous work has directly linked delphinidin to this specific immunological reprogramming. Given that CD8⁺CD122⁺ T cells exert robust suppressive functions and have been described as more potent regulators than classical CD4⁺CD25⁺ T_reg_ cells^37,45^, this finding uncovers a novel immunomodulatory property for anthocyanins.

Functionally, this immune restoration translated into improved forepaw use, preserved striatal dopaminergic terminals, and increased dopamine turnover in hαSYN^del^ mice. This rise in turnover most likely reflects a compensatory mechanism and a consequence of the surviving terminals, supporting improved motor performance despite ongoing dopaminergic cell loss. At the same time, such metabolic upregulation may imply increased oxidative demand, emphasizing the dual nature of this adaptation. Yet, delphinidin did not prevent dopaminergic cell loss in the SN. These findings indicate that delphinidin stabilized dopaminergic terminals and their functional dynamics rather than altering global dopamine metabolism or halting neurodegeneration in the SN.

However, pharmacokinetic considerations provide a possible explanation for our observation. Plasma measurements revealed a rapid peak and steep decline, with brain concentrations below the quantification limit of our assay. Under the once-daily dosing mandated by animal welfare regulations, insufficient central exposure likely constrained dopaminergic cell rescue despite clear terminal and immune effects.

Taken together, several limitations should be considered. Brain exposure to delphinidin remained below the limit of quantification, precluding firm conclusions about bioavailability. Especially since delphinidin was not detectable in the brain within our experimental setup, peripheral immune actions remain a plausible route for neuroimmune interactions orchestrated by delphinidin. However, our study was not designed to differentiate peripheral from central immune pathways, and we lack direct peripheral immune readouts; thus, any conclusions in this regard remain speculative. Future studies that combine detailed peripheral and central immune profiling will be required to clarify whether delphinidin acts primarily via peripheral immune modulation, direct CNS effects, or a combination of both. The once-daily injection paradigm of delphinidin also limited target engagement. The behavioral readouts focused mainly on the cylinder test, and we did not measure direct downstream markers of inflammasome activity (such as IL-1β) or regulatory cytokines (including IL-2, IL-10 and TGF-β). The absence of these readouts restricts in-depth mechanistic interpretation of the observed CD8^+^CD122^+^ enrichment and should be kept in mind when interpreting our findings. At the same time, these caveats help to define priorities for future work. From a translational perspective, our data suggest that increasing brain exposure through optimized formulation, dosing frequency, or prodrug strategies could enhance efficacy, and that CD8⁺CD122⁺ frequencies may serve as a practical biomarker to bridge preclinical and human studies in PD.

In conclusion, delphinidin mitigates microglial activation and reprograms adaptive immunity towards CD8⁺CD122⁺ regulation, showing functional improvement and terminal protection without dopaminergic cell rescue in the SN. The observed expansion of CD8⁺CD122⁺ T_reg_ represents the key immunological signature of delphinidin in α-synucleinopathy and highlights the potential of targeting regulatory CD8⁺ subsets in PD the future.

## Methods

### Animals

Healthy male C57Bl/6 J mice (Charles River, Sulzfeld, Germany) were kept in a near pathogen-free environment under standard conditions (21 °C, 12 h/12 h light– dark cycle) and provided chow and water ad libitum. To avoid possible hormonally induced bias regarding behavioral testing and the investigation of neurodegeneration, only male animals were used. In the experimental group, hαSYN mice were systemically treated with delphinidin (i.p.) (hαSYN^del^) for 9 weeks. Vehicle-treated hαSYN mice (hαSYN^veh^) and mice treated with empty AAV1/2-vector (EV), with (EV^del^) or without i.p. delphinidin treatment (EV^veh^) served as controls (Fig. 1).

### Adeno-associated vectors (AAV) 1/2 serotype injection

Custom made AAV1/2 (from GeneDetect Ltd (Auckland, New Zealand)) were designed, and experimental procedures were carried out as previously described^12^. In brief, 10-week-old deeply anaesthetized mice were placed into a stereotactic frame (Neurostar®) and injected unilaterally into the right SN with a microinjector (Stoelting, Kiel, Wisconsin, USA) and an injector-controlled 5 µl Hamilton syringe (Merck; cat. # 26200-U). The injection rate was 0.25 μl/min with either 2 μl of EV or 2 μl AAV1/2 carrying human mutated A53T-αSYN (hαSYN) with a concentration of 5.16 × 1012 genomic copies (gc) per ml. According to the mouse brain atlas of Paxinos and Franklin^46^ the following coordinates were used from Bregma: anterior-posterior (AP) −3.1 mm; medio-lateral (ML) −1.4 mm; dorso-ventral (DV) −4.4 mm. 70 mice were injected with AAV1/2-A53T-α-Synuclein (hαSYN), 60 mice with AAV1/2-EV (EV).

Four groups of mice were included in the study: hαSYN or EV mice were treated daily with either 50 µg/kg BW of delphinidin (complexed with SBECD; obtained from: CYCLOLAB Ltd. (Budapest, Hungary), RRID: Batch Number: CYL-4000) or an equal amount of SBECD (obtained from: CYCLOLAB Ltd. (Budapest, Hungary), Batch Number: CYL-3561; vehicle) i.p., respectively, diluted in 0.9% sterile physiological sodium chloride solution directly before application. 50 µg/kg BW delphinidin-SBECD corresponds to 1 µg/kg BW of delphindinchloride. Supplementation with i.p. delphinidin or vehicle treatment was initiated with a latency of 7 days after AAV1/2 transfection (Fig. 1).

### Behavioral studies - cylindertest

Spontaneous forepaw use was assessed using the cylinder test before and 9 weeks after the EV or hαSYN injection (Fig. 1), as previously described^3,12,25^.

### Tissue processing and immunohistochemistry

Mice were sacrificed 10 weeks after viral vector injection by perfusion with 0.1 M phosphate-buffered saline (PBS). For high-performance liquid chromatography (HPLC) analysis fresh mouse brains were processed further, dissecting the striatum in coronal plane in a ventral and dorsal part (Figs. 30–27 [Bregma + 0.14 mm] and Figs. 25–23 [Bregma + 0.74 mm] according to Paxinos and Franklin^46^). The ventral part was snap frozen in liquid dry ice-cooled isopentane for HPLC analysis. The dorsal part was immersionfixed in 4% Paraformaldehyde (PFA) in 0.1 M PBS for 2 days and then rinsed in 30% sucrose/0.1 M PBS for another 2 days, followed by freezing of the tissue in liquid dry ice-cooled isopentane. After embedding in Tissue-Tek® (O.C.T Compound Sakura), the dorsal part (Bregma −2.06 to −4.04 mm) was serially cut into 40 μm-thick coronal sections on a cryostat (Leica 3050, Leica Biosystems). One series (out of five) was used for tyrosine hydroxylase (TH) and Nissl double staining. After three washing steps with 0.1 M PBS, sections were treated with blocking solution (10% normal goat serum (NGS), 2% bovine serum albumin (BSA), 0.5% Triton X-100 (SigmaAldrich, cat. #X100) in 0.1 M PBS (PBT) for 1 h. The primary antibody (rabbit anti-tyrosine hydroxylase; Abcam, cat. #ab112; RRID:AB_297840, 1:1000) was incubated in 2% NGS, 2% BSA in PBT, overnight at room temperature (RT). As a secondary antibody, biotinylated goat anti-rabbit (Vector Laboratories, cat. #BA-1000; RRID:AB_2313606) was used at 1:100 dilution in 2% NGS, 2% BSA in PBT, for 2 h at RT. After 2 h incubation in an avidin/biotin solution (Standard Ultra-Sensitive ABC Staining Kit (32050), Thermo Fisher Scientific), the sections were developed with diaminobenzidine (DAB)-HCl and H_2_O_2_ (Peroxidase Kit, Vector Laboratories, cat. #SK-4100). Sections were incubated in cresyl violet solution (1 g of cresyl violet + 10 ml of 100% acetic acid and 1 L of distilled water; Certistain®, SigmaAldrich, cat. #105235) for 30 min at RT to finalize Nissl staining.

For immunohistochemical staining of brain tissue for CD4^+^, CD8^+^, CD11b^+^ cells, and TH^+^ striatal fibers, brains were snap frozen. The striatum and SN were serially cut into 10 µm-thick coronal sections. Stainings of the striatum were performed at +0.14 mm from Bregma according to Paxinos and Franklin^46^.

After fixation with either acetone for T cell staining or 4% PFA for labeling of myeloid cells and TH^+^ fibers for 15 min. sections were blocked specifically for later staining (TH fibers [2% BSA and 10% NGS], CD4/8 [5% BSA], CD11b [1% BSA, 5% NGS]). Subsequently, sections were incubated overnight with rat anti-mouse CD4 (1:1000, Bio-Rad, cat. #MCA1767; RRID:AB_322769), rat anti-mouse CD8 (1:500, Bio-Rad, cat. #MCA609G; RRID:AB_321407), rat anti-mouse CD11b (1:100, Bio-Rad, cat. #MCA711; RRID:AB_321292), or rabbit anti-TH antibodies. Biotinylated rabbit anti-rat or goat anti-rabbit antibodies were used (Vector Laboratories, cat. #BA-4001; RRID:AB_10015300 and BA-1000; RRID:AB_2313606) as secondary antibodies. DAB staining was performed as described above.

### Immunofluorescence stainings

Immunofluorescence (IF) stainings of TH, CD11b, and DAPI (4′,6-diamidino-2-phenylindole) on fresh-frozen sections were performed as described before^12^. In brief, 40 µm interval-sections of the SN were blocked with 10% NGS and 2% BSA for 2 h at RT. Primary antibodies (chicken anti-mouse TH; abcam cat. #76442; RRID: AB_1524535, 1:500; rat anti-mouse CD11b) were incubated in a humidified chamber overnight at 4 °C. Secondary antibodies (anti-chicken AF488, Invitrogen cat #A11039; RRID: AB_253409; goat anti-rat AF647, abcam cat. #150167; RRID:AB_286429); 1:300 each) were incubated in a humidified chamber for 2 h at RT.

For TH, CD8, and CD122 staining on 10 µm fresh frozen sections of the SN were blocked with 5% NGS and 5% BSA for 1 h at RT. Primary antibodies (chicken anti-mouse TH; abcam cat. #76442; RRID: AB_1524535, 1:500; rat anti-mouse CD8; Bio-Rad cat. #MCA609G; RRID: AB_321407, 1:500; rabbit anti-CD122, MyBioSource cat. #MBS8241964; RRID: N/A, 1:150) were incubated in a humidified chamber at 4 °C overnight (1% BSA, 1% NGS). Secondary antibodies (goat anti-rat AF647, abcam cat. #ab150167, RRID:AB_2864291, 1:300; goat anti-rabbit AF488, Invitrogen cat. #A1-11008, RRID: AB_143165, 1:300; goat anti-chicken Cy3, Alpha Diagnostics cat. #60334, RRID: N/A, 1:300) were incubated in a humidified chamber for 2 h at RT. Finally, slices were mounted with DAPI-mounting solution (Invitrogen cat. # 00-4959).

### Stereological quantification of TH^+^ neurons

For analysis of TH+ neurons in the SN pars compacta (SNpc), a Stereo Investigator software package (version 11.07; MicroBrightField Biosciences, Williston, VT, USA) was used. The investigator was blinded to the individual animals and groups during counting. Settings were chosen as described^12^. The hemisphere ipsilateral to the viral vector injection was analyzed. 40 µm sections in 200 µm intervals, representing the entire SN, were used for cell counting. Sections were analyzed with a 100X/1.25 numerical aperture objective on a BX53 microscope (Olympus). The counting parameters were: grid size 110 × 110 μm; counting frame 50 × 50 μm; guard zone 2 μm zone. A Gundersen coefficient of error (for m = 1) of <0.1 was accepted.

### Analysis of immunohistochemical stainings of the brain

Immune cell analysis of IHC stainings was performed as recently described^12^. CD4^+^ and CD8^+^ T cells were quantified manually using a BH2 light microscope (Olympus) and a 64X objective. The area of the structure was calculated using NIH ImageJ (“Fiji” version 2.0-rc-69/1.52p, RRID:SCR_002285). CD11b^+^ cells of the SN were also counted manually using a 40X objective and a counting grid. The optical density (OD) of the DA terminals in the striatum was determined as previously described^25^. The OD of the corpus callosum was used as background. For all procedures described, images were acquired in 16-bit with an Axio Imager.M2 (20X, air). For presentation in the paper figures images were captured with Glissando whole-slide scanner (40X, Objective Imaging).

### Analysis of immunofluorescence stainings of the brain

Images were acquired using the Axio Imager M2 upright microscope equipped with ApoTome (10X, air). Quantification was performed on these images following a semi-automated workflow.

Cell counting was conducted as follows: Images were processed using ImageJ (version 1.53f51). First, the CZI files were opened, and the individual channels were split. A specific region of interest (ROI) was defined in the TH channel and saved in the ROI manager. This ROI was then applied to the CD8, CD122, and DAPI channels. The channels were subsequently merged, and areas of interest were examined at higher magnification to enable accurate manual cell counting.

### Analysis of bioavailability by liquid chromatography tandem mass spectrometry

A sensitive liquid chromatography tandem mass spectrometry (LC-MS/MS) method was developed to determine delphinidin in mouse plasma and brain tissue. Blood samples were collected in heparin-coated tubes and centrifuged immediately after withdrawal. To stabilize 200 µL of heparin plasma, 20 µL of 1.0 M HCl was added, followed by 800 µL of 0.1 M HCl in methanol for protein precipitation. Samples were vortexed and maintained on ice. After centrifugation at 21,130 rcf for 10 min at 4 °C, the supernatant was collected for LC-MS/MS analysis. Quantification was performed using an external standard method, with calibration solutions prepared by spiking delphinidin into a matched blank matrix. Accurately weighed brain tissue (0.4–0.5 g) was immediately stabilized with 200 µL of 1.0 M HCl and homogenized. For extraction, 500 µL of 0.1 M HCl in methanol containing 0.5% Triton X-100 was added, followed by vortexing and incubation on ice. After centrifugation at 21,130 rcf for 10 min at 4 °C, 200 µL of the supernatant was mixed with 20 µL of 1.0 M HCl, centrifuged, and processed for LC-MS/MS analysis. Quantification was performed using an external standard method, with calibration solutions prepared by spiking delphinidin into a matched blank matrix.

All chromatographic solvents were LC-MS grade. Delphinidin-SBECD complex was purchased from CYCLOLAB Ltd. (Budapest, Hungary), Batch Number: CYL-4000 (content: 2.00% delphinidin chloride). Chromatographic separation was carried out on a Kinetex C18 100 Å (100 mm × 2.1 mm, 2.6 μm) column (Phenomenex), at 50 °C with a mobile phase flow rate of 0.4000 mL/min. Mobile phases of 1.0% formic acid in water (MP-A) and 1.0% formic acid in a mixture of 70 vol.% methanol and 30 vol.% acetonitrile were used. Chromatographic separation was performed using a gradient as follows: 0–0.30 min, 5% MP-B; 0.30–0.40 min, 5–17% MP-B; 0.40–2.90 min, 17–37% MP-B; 2.91–3.30 min, 90% MP-B; 3.31–4.30 min, 5% MP-B as equilibration time. Injection volume was 4 μL and the samples were kept at 20 °C throughout the analysis. The mass spectrometric analysis was performed using a Shimadzu 8030^+^ triple-quadrupole mass spectrometer coupled with an ESI source in the positive ion mode. Multi reaction monitoring (MRM) mode was applied for quantification using target fragment ions m/z 302.90 → 229.00 for delphinidin. The previously optimized interface settings were as follows: Nebulizing Gas Flow 1.4 L/min; Drying Gas Flow 3.0 L/min; Desolvation Line Temperature 125 °C; Heat Block Temperature 500 °C; Interface Voltage 0.7 kV.

### High-performance liquid-chromatography

High-performance liquid chromatography was carried out as described earlier^47^. After organ harvesting, both striata were snap-frozen in liquid nitrogen and homogenized in 150 mM H_3_PO_4_ and 500 μM diethylenetriaminepentaacetic acid (Sonopuls Ultrasonic homogenizer (Bandelin, Berlin, Germany)). Homogenized striata were centrifuged with 20,817 g for 15 min. Afterwards, 5 μl of the supernatant were transferred to a Nucleosil 120–5 C18 column (250 mm × 4 mm, 5 μm; Macherey-Nagel, Düren, Germany). The mobile phase was composed of 0.975 mM octanesulphonic acid, 0.5 mM triethylamine, 0.1 mM ethylenediaminetetraacetic acid, 2 mM KCL, 0.09 M Na_2_HPO_4,_ and 14% methanol (pH adjusted to 3.88 with phosphoric acid). Recordings were performed at 37 °C and with an electrode potential of +0.77 V versus the Ag/AgCl reference electrode using the Agilent 1260 LC System measurements. 2,3-Dihydroxybenzoic acid was used as an internal standard. We analyzed the striatum for dopamine (Dopa), homovanillic acid (HVA), 3,4-dihydroxyphenylacetic acid (DOPAC), serotonin (5-HT) and 5-hydroxyindoleacetic acid (5-HIAA). An external standard calibration was used for quantification. The amount of total protein was determined by the Lowry reagent and the results were normalized to neurotransmitter levels found in control animals (EV^veh^).

### Statistical analysis

For statistical analyses, Graph Pad Prism Version 10.2.3 was used. Normality was determined by the Shapiro–Wilk test. Normal distributed data sets were statistically analyzed using one-way ANOVA and the Tukey’s multiple comparison test. Non-parametric data was analyzed using the Kruskal–Wallis test followed by Dunn’s posttest. The graphs indicate mean ± standard error of the mean (SEM). (*) P <0.05, (**) P <0.01, (***) P <0.001, and (****) P <0.0001 were considered significant p values.

### Ethics statement

All experiments were performed in accordance with the guidelines of the European Union and approved by our institutional Animal Care the Utilization Committee and the Regierung von Unterfranken, Würzburg, Germany (License number: RUF-55.2.2-2532-2-522). The Regierung of Unterfranken Würzburg approved all animal experiments.

## Data Availability

The datasets generated and/or analyzed during the current study are not publicly available due to internal data governance policies and the technical complexity of the raw animal data, but are available from the corresponding author on reasonable request.
